# Modeling the mechanical stiffness of pancreatic ductal adenocarcinoma

**DOI:** 10.1016/j.mbplus.2022.100109

**Published:** 2022-03-21

**Authors:** Delanyo Kpeglo, Matthew D.G. Hughes, Lorna Dougan, Malcolm Haddrick, Margaret A. Knowles, Stephen D. Evans, Sally A. Peyman

**Affiliations:** a Molecular and Nanoscale Physics Group, School of Physics and Astronomy, University of Leeds, LS2 9 JT, UK; b Astbury Centre for Structural Molecular Biology, University of Leeds, LS2 9JT, UK; c Leeds Institute of Medical Research at St James’s (LIMR), School of Medicine, University of Leeds, LS2 9 JT, UK; d Medicines Discovery Catapult, Block 35, Mereside Alderley Park, Alderley Edge, SK10 4TG, UK

**Keywords:** Tissue mechanics, Tumor microenvironment, Pancreatic ductal adenocarcinoma, Pancreatic stellate cells, Transforming growth factor β1, Oscillatory shear rheology, Tumour biophysics

## Abstract

Despite improvements in the understanding of disease biology, pancreatic ductal adenocarcinoma (PDAC) remains the most malignant cancer of the pancreas. PDAC constitutes ∼95% of all pancreatic cancers, and it is highly resistant to therapeutics. The increased tissue rigidity, which stems from the rich fibrotic stroma in the tumor microenvironment, is central to disease development, physiology, and resistance to drug perfusion. Pancreatic stellate cells (PSCs) are responsible for overproduction of extracellular matrix in the fibrotic stroma, and this is exacerbated by the overexpression of transforming growth factor-β (TGF-β). However, there are few *in vitro* PDAC models, which include both PSCs and TGF-β or mimic *in vivo*-like tumor stiffness. In this study, we present a three-dimensional *in vitro* PDAC model, which includes PSCs and TGF-β, and recapitulates PDAC tissue mechanical stiffness. Using oscillatory shear rheology, we show the mechanical stiffness of the model is within range of the PDAC tissue stiffness by day 21 of culture and highlight that the matrix environment is essential to adequately capture PDAC disease. PDAC is a complex, aggressive disease with poor prognosis, and biophysically relevant *in vitro* PDAC models, which take into account tissue mechanics, will provide improved tumor models for effective therapeutic assessment.

## Highlights

• The PDAC stroma stiffness underlines its malignant behavior and drug resistance.
• 3D *in vitro* cultures must model the PDAC stroma to effectively drug efficacy.
• PSCs are responsible for the stroma, and its activity is increased with TGF-β.
• Develop a 3D culture model of PDAC, which includes PSCs and TGF-β.
• Assess the mechanical stiffness, stain for collagen, and investigate gemcitabine efficacy.

## Graphical abstract

Tumor tissue stiffening drives tumor development, physiology, and poor drug perfusion. Tumor stiffness in PDAC is central to the disease aggressiveness, chemotherapeutic resistance, and the poor prognosis observed. It is important 3D *in vitro* models reflects the microenvironment and mechanical characteristic of the PDAC tissue to accurately develop and assess potential therapeutics and improve patient survival.

**Figure:**
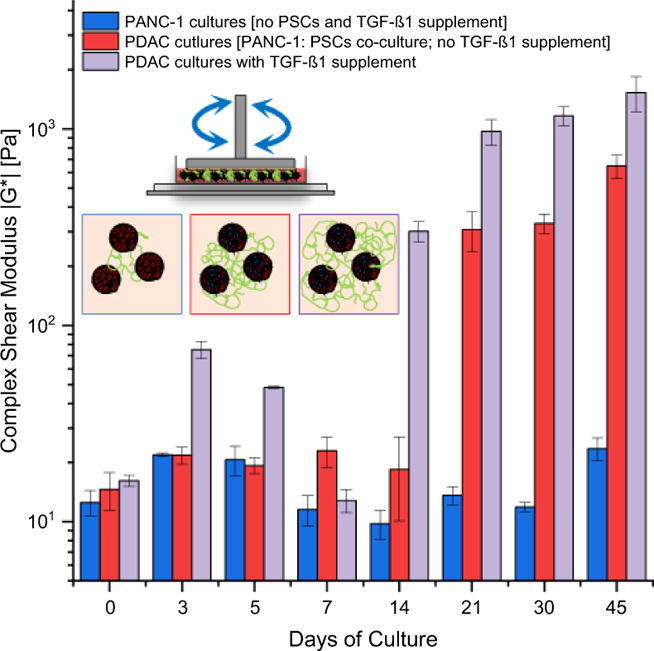

## Introduction

Pancreatic ductal adenocarcinoma (PDAC) is the most malignant pancreatic cancer, with a 5-year survival rate of ≤9% [1], [2], [3], [4]. It is commonly diagnosed during its advanced stages, when surgical excision may not be an option due to the involvement of blood and lymphatic vessels and the performance status of the patient [4], [5], [6], [7]. Chemotherapy at these later stages is also extremely limited in effectiveness, due to a number of factors including poor drug penetration into the tumor tissue [6], [8], [9].

PDAC exhibits a dense, fibrotic stroma, which contributes to the poor clinical outcome of the disease by providing a physical barrier between the cancer cells and incoming therapeutics [6], [8], [9]. The dense consistency of the PDAC stroma is a result of the overproduction of extracellular matrix (ECM) macromolecules by the presence of pancreatic stellate cells (PSCs) [3], [8], [10], [11], [12]. These fibroblast cells have a symbiotic relationship with pancreatic cancer cells and are responsible for the regulation of the ECM. In healthy pancreatic tissue, in the absence of cancer cells, PSCs are reported to be quiescent [8], [10], [13], [14], [15]. However, in the presence of the cancer cells, PSCs become activated via signaling pathways, such as the mitogen-activated protein kinase (MAPK) and SMAD [8], [10], [12], [13], [15] and produce copious amounts of ECM macromolecules, which includes fibrillar type I collagen and the glycosaminoglycan, hyaluronan (HA), mediated by cytokines such as TGF-β1, creating a tumor progressive environment [9], [10], [12], [14], [16], [17], [18], [19], [20], [21], [22]. The over secretion of these ECM macromolecules produces a rigid matrix, which can account for 90% of the tumor volume [9], [18], [21], [23], [24].

The rigid stroma promotes tissue tensional homeostasis, and the PDAC cancer cells respond by altering their mechanical phenotype with high levels of cell contractility to counteract the stiff environment [9], [23], [24], [25], [26]. This sets off a positive feedback cycle of matrix production, and signaling pathways, including TGF-β, are induced to exacerbate the growth of PDAC's fibrotic stroma and tissue stiffness [3], [10], [18], [24], [27].

The resultant increase in the matrix stiffness can deform and even collapse surrounding tissue structures such as blood and lymphatic vessels, which limits the flow of nutrients, oxygen, and therapeutics to the cancer cells [3], [6], [9], [28]. Therefore, the PDAC stroma, with changes in its mechanics, contributes to the disease’s malignancy, progression, and therapeutic resistance [3], [9], [21], [23].

Studies on PDAC, commonly with two-dimensional (2D) cultures or mouse models, have established that tissue stiffening is a hallmark of the disease state, which precedes and drives tumor progression [3], [23], [26], [28], [29], [30], [31], [32]. However, 2D models do not adequately recapitulate the three dimensional (3D) hierarchical complexities of the PDAC tissue, and mouse models do not accurately predict the drug pharmacodynamics observed in humans [33], [34], [35]. 3D spheroid and organoid cultures have helped to model the 3D complexities of these tumors, facilitating cell–cell and cell-matrix interactions, with studies elucidating the role of PSCs and ECM macromolecules on the homeostasis of the PDAC tumor microenvironment and its malignancy [22], [33], [34], [35], [36], [37], [38]. Imaging and immunohistochemistry studies of these 3D culture models, (and of mice and human PDAC tissues) have shown that the production and accumulation of macromolecules in the ECM is associated with therapeutic resistance [17], [22], [30], [31], [34], [38]. Despite this, current 3D culture models still fail to investigate or mimic PDAC tumor stiffness, which is a great concern given that the rigidity associated with the fibrotic stroma (>1 kPa) accounts for chemotherapeutic resistance [23], [31], [38], [39], [40], [41]. Thus, mechanically relevant models are needed to reflect PDAC’s stiffness to better assess current and new therapeutics.

Here, the PDAC cancer cell line, PANC-1, was cultured with PSCs and TGF-β1 supplement, and the growth and mechanical stiffness of the cultures was assessed with oscillatory shear rheology. We show that by day 21, the stiffness of the PANC-1 with PSCs and TGF-β1 was within range of the PDAC tissue mechanical stiffness [23], [26], [32]. To the best of our knowledge, shear rheology has been used to measure the mechanical properties of healthy and diseased *ex-vivo* pancreatic tissues [32] but has not been applied to *in vitro* pancreatic culture models. Oscillatory shear rheology was applied to assess the matrix and cellular dynamics of PANC-1 cultures in the presence of PSCs and TGF-β with increasing culture time [32], [42], [43], [44] in order to develop an *in vitro* PDAC model that reflects the mechanical stiffness of the tissue. Once this was achieved, the effect of gemcitabine was assessed. We highlight the importance of having a relevant PDAC model, which takes into account the cell types and disease stiffness absent in current 3D PDAC models.

## Results

### PSC cells increase PANC-1 spheroid growth

In order to assess the effect of PSCs on PANC-1 spheroid growth, the size and doubling time of PANC-1 spheroids were measured with and without PSCs. The PANC-1 cells were seeded with PSCs into ULA plates for spheroid culture and allowed to grow for 14 days or 21 days. The spheroid cultures maintained for 21 days were used to investigate the long-term effect of the PSCs on the PANC-1 spheroid growth. Due to the hydrophilic coating of the ULA plates, the cells are forced into a suspended state, enhancing cell–cell interactions over cell-substrate interactions. Images were taken daily to monitor the growth of the spheroids by evaluating their diameter over the culture period. Their volume was also assessed to determine spheroid doubling time, the time it takes for the spheroid size to double in volume.

**Fig. 1:**
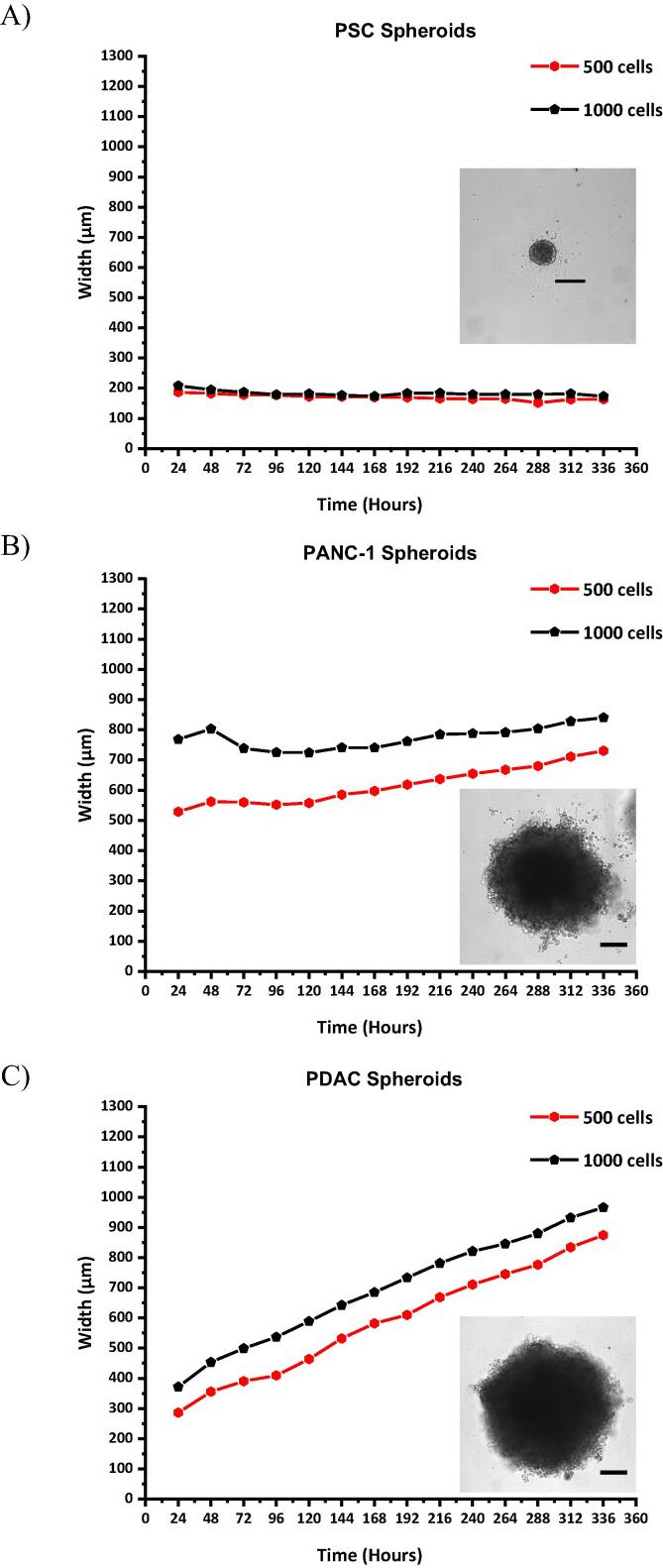
Spheroid diameter (width), of the A) PSC only spheroids, (B) PANC-1 only spheroids, and C) PDAC spheroids (PANC-1 and PSCs co-culture, 1:2 seeding ratio) over the 14-day culture period, with their respective bright field images on day 14 of culture. Scale bar, 200 µm.

Fig. 1 shows spheroid diameter over time in hours for two cell seeding densities, 500 and 1000 cells per well, for PSC only spheroids, PANC-1 only spheroids and PDAC spheroids. The cultures of PSC only cells (Fig. 1A) did not show significant growth regardless of seeding density, which suggests they were dormant as spheroids over the 14-day culture period. The PANC-1 only spheroids (Fig. 1B) showed an increase in diameter, starting at widths of approximately 500 µm and 700 µm to 700 µm and 800 µm for seeding densities of 500 and 1000 cells per well, respectively. Spheroid growth rate of the PDAC spheroids (Fig. 1C and Fig. S1) was significantly higher compared to PSC and PANC-1 only spheroids (Fig. 1A and B, respectively). After 24 h, PDAC spheroid diameters were 286 µm and 370 µm, and after 14 days, they had grown to 870 µm and 960 µm, for 500 and 1000 cell seeding densities, respectively (Fig. 1C). For the PDAC spheroids supplemented with TGF-β1 (10 ng mL^−1^), the diameter was 800 µm by day 14 of culture for a seeding density of 250 cells per well (Fig. S1B). However, the spheroid growth decreased between day 14 and 21 of culture, similar to the avascular growth phase of solid tumors [45], [46]. The mean doubling time of the PDAC spheroids, irrespective of seeding density, or seeding ratio between the PANC-1 and PSC, was approximately 3 days (Table S1).

### PDAC spheroids contain regions in different proliferative states

**Fig. 2:**
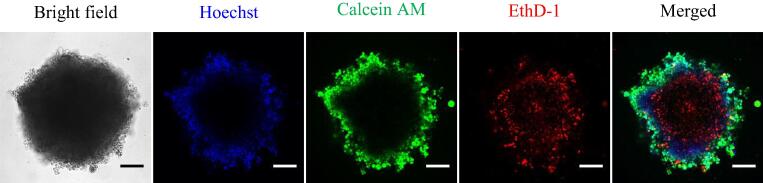
Live/dead viability assessment of the PDAC spheroids (seeding density of 1000 cell per well; seeding ratio of PANC-1 and PSC co-culture, 1:2) on day 14 of culture with Hoechst, Calcein AM, and EthD-1 viability stains. Scale bar, 200 µm.

A characteristic of spheroids is their ability to mimic the spatial or zonal regions of the physiological environment of solid tumors with respect to nutrient availability and oxygen gradients [38], [47]. The zonal regions of the physiological environment of solid tumors are well established in the literature, but we show here our cultures exhibit these regions and reflect the physiological environment of the PDAC tissue as a solid tumor. To observe these different zones in our cultures, the PSC only, PANC-1 only, and PDAC (co-culture of the PANC-1 and PSC cells at a seeding ratio of 1: 2 and 1:3) spheroids, live/dead viability assessments were performed to determine their metabolic state using Hoechst, Calcein Am, and EthD-1 (Figs. 2 and S2). Hoechst binds to the cell nucleus of live cells to fluoresce blue, Calcein AM is enzymatically converted to the fluorescent green calcein in live cells, and EthD-1 binds to the nucleic acid of dead cells to fluoresce red. The spheroids exhibited an outer region of proliferative cells (Calcein AM stain, green), where the cells have enough nutrients to undergo cell division, a region of quiescent cells (Hoechst stain, blue), where the cells are alive but do not have enough nutrients and oxygen for cell division, and a central region of dead cells (EthD-1 stain, red), where there is a very limited supply of nutrients and oxygen. The PDAC spheroids, cultured for 21 days, were also assessed with Hoechst, Calcein AM, and EthD-1, and they also exhibited cells with the stratified metabolic regions (Fig. S2). Assessment of their metabolic activity over the 21-day culture period (Fig. S3, an extra data point day 28 was also included) showed the spheroids were viable.

### PDAC cultures increase in mechanical stiffness with TGF-β1

**Fig. 3:**
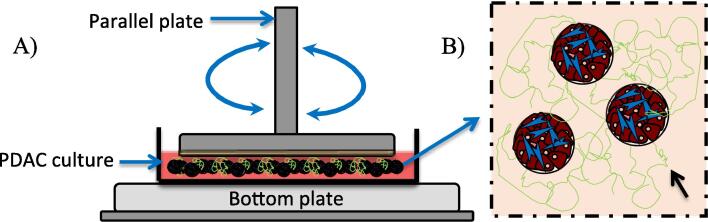
Schematic of the shear deformation of the PDAC cultures. A) An illustration of the use of the parallel plate rotary system for oscillatory shear deformation of the PDAC cultures grown in a culture petri dish to determine their storage (*G′*) and loss (*G″*) moduli. B) An illustration of the PDAC cultures with a matrix environment, top view. Black arrow indicates a depiction of collagen observed in the PDAC culture environment.

**Fig. 4:**
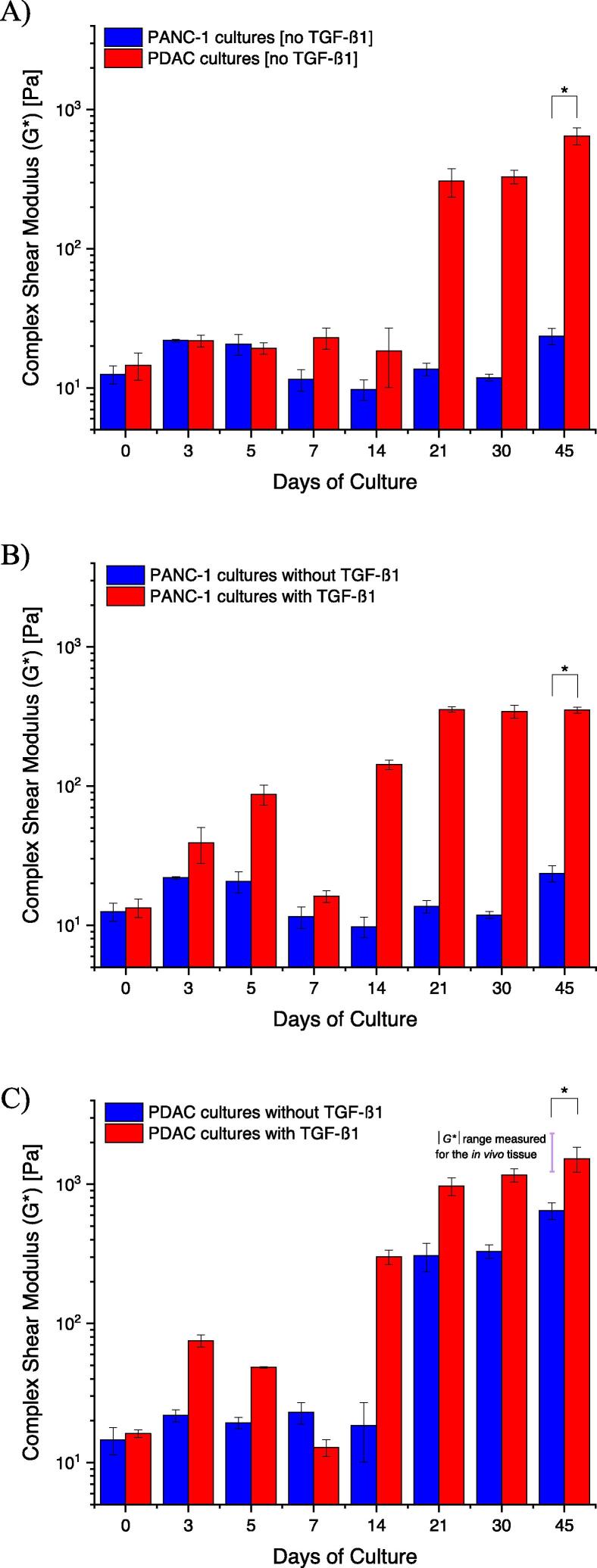
The complex shear modulus (|G*|), mechanical stiffness, of PANC-1 only and PDAC (PANC-1 and PSC co-culture at a seeding ratio of 1:3) cultures with and without TGF-β1 (10 ng mL^−1^) growth factor supplement. A) PANC-1 only cultures and PDAC cultures without TGF-β1, B) PANC-1 only cultures with and without TGF-β1, and D) PDAC cultures with and without TGF-β1. Data are mean ± SE with a *p-value < 0.05, where *n* = 9 culture plates per culture condition generated from three separate seeding settings.

In order to investigate the effect of both PSCs and TGF-β on the mechanical stiffness of the cultures, PANC-1 cells were seeded with and without PSCs into culture dishes with BME gel as an ECM. The PANC-1 only cultures and PDAC (PANC-1 with PSC co-culture in a 1:3 seeding ratio) cultures were maintained for 45 days with medium supplemented with TGF-β1 growth factor (10 ng mL^−1^). The mechanical stiffness of the PANC-1 only cultures and PDAC cultures, with and without TGF-β1 over the 45-day culture period, was assessed with measurements of their *G′* and *G″* moduli components using oscillatory shear deformation (Fig. 3). Steady state values of *G′* and *G″* were extracted from the time sweep curves (Figs. S4 and S5, exemplar plots of *G′* and *G″* measurements, and extracted values, respectively) and used to determine |G*| for the different culture conditions A) PANC-1 only cultures and PDAC cultures, B) PANC-1 only cultures with and without TGF-β1 supplement, and C) PDAC cultures with and without TGF-β1 supplement (Fig. 4). The mechanical stiffness of the PSC cultures with and without TGF-β1 growth factor were not assessed as the PSCs in the PSC only cultures were believed to be dormant (Fig. 1A and Fig. S1B).

PANC-1 only and PDAC cultures, in the absence of TGF-β1 supplement, were initially observed to have a |G*| of approximately 13 Pa (±2) and 15 Pa (±3), respectively (Fig. 4A). After 45 days of culture, the PANC-1 only cultures were measured to have a |G*| of 23 Pa (±3), and the PDAC cultures, a |G*| of 650 Pa (±9) (Fig. 4A). With TGF-β1 growth supplement, the PANC-1 only cultures increased to a |G*| of 350 Pa (±18) on day 45 of culture (Fig. 4B). However, after 21 days, the PDAC cultures, supplemented with TGF-β1, exhibited a |G*| of 970 Pa (±143) (Fig. 4C), a value corresponding to a Youngs modulus (*E*) of 2.9 kPa (using Eq. (3)) and approaching the mechanical stiffness measured for the PDAC tissue [23], [32]. On day 45 of culture, the PDAC cultures with TGF-β1 showed an increase |G*| to approximately 1500 Pa (±310) (Fig. 4C). A decrease in |G*| between day 5 and 14 (Fig. 4A–C) was observed, believed to be effects of the cells remodeling the matrix with the secretion of enzymes and proteases [9], [18], [21], [22]. Frequency sweep measurements of the cultures (Fig. S8), to assess their time-dependent behavior, show the cultures were stable during shear deformation. Fig. S6 shows examples of the range of normal forces measured by the parallel plate rotary system to ensure contact with the cultures during deformation.

We evaluated whether the measured mechanical stiffness was a result of the cells and spheroid number and the relative area occupied by the cells, spheroids, and BME gel. Brightfield images of the cultures were taken, and Fig. S7 shows the relative area occupied by the cells and spheroids versus BME gel and the ECM of the cultures, did not differ between the different culture conditions, and that the complex shear modulus measured (Fig. 4) for the different culture conditions was not due to the ratio of cells, which increased with increase culture time consistently between culture conditions. Fig. S9 shows the |G*|, *G′* and *G″,* and frequency sweep measurements of just BME gel over a 21-day period. An overall decrease in |G*|, over the 21-day period, further demonstrates that the increase in the mechanical stiffness of the PANC-1 cultures with PSCs, and TGF-β1 supplement, was as a result of the cells creating an environment beneficial to their growth.

**Fig. 5:**
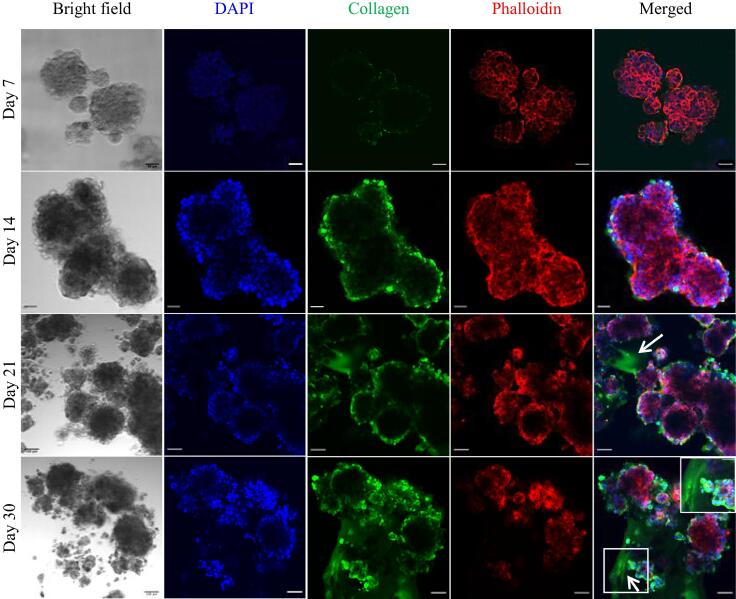
Immunofluorescence stain of collagen type I of the PDAC (PANC-1 and PSC co-culture at a seeding ratio of 1:3) cultures with TGF-β1 on day 7, 14, 21 and 30 of culture. Nuclei stained with DAPI in blue, collagen in green, actin with phalloidin in red. Box magnification on day 30 show collagen fibers. Scale bar on day 7 and 14, 50 µm. Scale bar on day 21 and 30 of culture, 100 µm. (For interpretation of the references to colour in this figure legend, the reader is referred to the web version of this article.)

To assess the production of ECM proteins by the cultures, the PANC-1 only and PDAC (PANC-1 with PSCs in a 1:3 seeding ratio) cultures were fixed and stained for collagen type I on day 7, 14, 21, and 30 of culture. This was performed to determine if the increase in mechanical stiffness was due to the presence of collagen. The PSC only cultures were also fixed and stained for collagen type I, but no collagen was observed. In the PANC-1 only cultures with and without TGF-β1, there was no evidence of collagen in the matrix environment (Figs. S10 and S11), but collagen was observed to be localized at the culture periphery. In the PDAC cultures without TGF-β1, collagen was mostly observed in the culture periphery (Fig. S12). With TGF-β1, collagen was observed in the matrix environment of the PDAC cultures by day 21 of culture and was extensive on day 30 of culture (Fig. 5). This shows with the presence of PSCs and TGF-β1, there is the overproduction of collagen accounting for the mechanical rigidity [3], [12], [18], [21], observed for the PDAC cultures (Fig. 4A–C) and reflected in their increased elasticity and viscosity (Fig. S5). The increase in elasticity and viscosity demonstrates the ability of the cells to adapt and respond to the growing mechanical forces of their matrix [3], [26], [40], [42].

### Effect of gemcitabine on the PDAC cultures

**Fig. 6:**
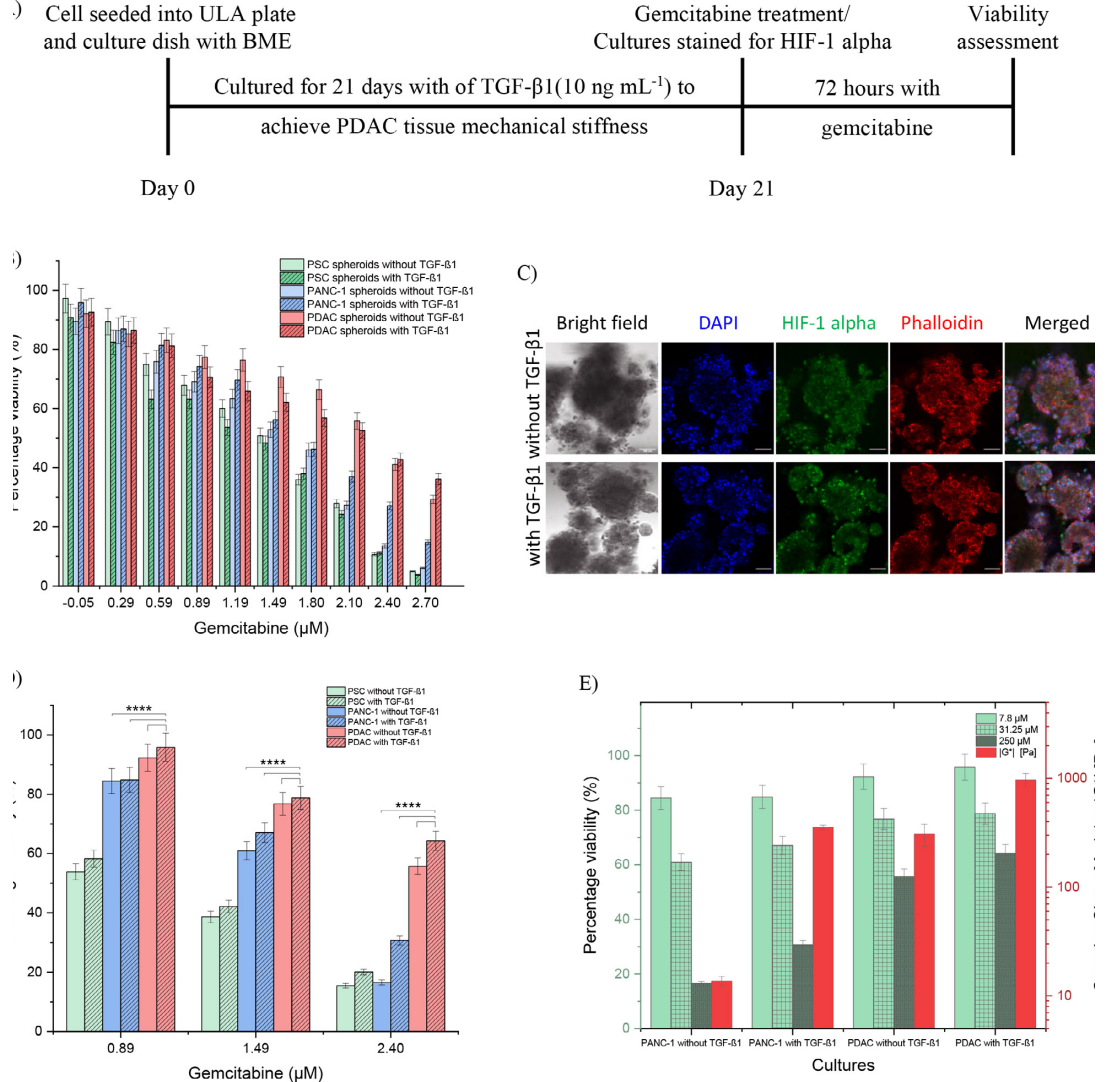
Gemcitabine effect on the PSC only, PANC-1 only, and PDAC (PANC-1 and PSC co-culture at a seeding ratio of 1:3) cultures with and without BME gel and TGF-β1 (10 ng mL^−1^) growth factor supplement. A) Timeline of the seeding of cells for culture to treat with gemcitabine. B) The percentage viability of the spheroids versus the log scale of the gemcitabine concentrations. A total of *n = 12* spheroids per culture condition generated from two separate seeding settings. C) The PDAC cultures with and without TGF-β1 stained for HIF-1 alpha on day 21 of culture. Nuclei stain with DAPI (blue), and actin with phalloidin (red). D) The percentage viability assessment of the cultures versus the log scale of the gemcitabine concentrations, 7.8, 31.25, and 250 µM. E) The percentage viability versus the different culture conditions and their corresponding complex shear modulus measured in Fig. 4. A total of *n* = 4 culture plate per culture condition generated from two separate seeding settings. ****p < 0.0001, two-way ANOVA followed by Tukey’s multiple comparison test. (For interpretation of the references to colour in this figure legend, the reader is referred to the web version of this article.)

With *in vivo-*like PDAC tissue mechanical stiffness achieved by day 21 of culture, PANC-1 and PSC cells were seeded into ULA plates for PSC only, PANC-1 only, and PDAC (PANC-1 and PSC co-culture at a seeding ratio of 1:3) spheroids and maintained for 21 days with and without TGF-β1 (10 ng mL^−1^) supplement. The spheroids were then treated with gemcitabine at different concentrations for 72 h and their viability assessed. Fig. 6A shows the timeline of the seeding of cells and gemcitabine treatment, and Fig. 6B shows the percentage viability (normalized to controls) versus the different concentrations of gemcitabine used, in log scale, for the different culture conditions.

There was a decrease in percentage viability for the different culture condition with an IC_50_ of 1.7 (±0.08), 2.1 (±0.09), and 2.1 (±0.13) for the PANC-1 only spheroids with TGF-β1 and the PDAC spheroids with and without TGF-β1, respectively. The PDAC spheroids with and without TGF-β1 were more resistant to the effects of gemcitabine in comparison to the PANC-1 cultures with and without TGF-β1, and this may have been the effect of how rigid the cultures were as this limits how drugs, here gemcitabine, penetrate through to the cells [3], [25], [45], [48], [49].

To further assess how the dense and stiff cultures affect gemcitabine efficacy, PANC-1 and PSC cells were seeded into the culture dishes with BME gel for PSC only, PANC-1 only, and PDAC (PANC-1 and PSC co-culture at a seeding ratio of 1:3) cultures and maintained for 21 days with and without TGF-β1 (10 ng mL^−1^). The cultures were then treated with 7.8, 31.25 and 250 µM gemcitabine and their viability assessed. The cultures were also stained for hypoxia-inducible factor-1 alpha (HIF-1 alpha) to determine if they were hypoxic. Fig. 6A shows the timeline for the seeding of cells and gemcitabine treatment, and Fig. 6C show images of the HIF-1 alpha stain for the PDAC cultures with and without TGF-β1 on day 21 of culture. Fig. 6D shows the percentage viability (normalized to controls) versus the gemcitabine concentrations, in log scale, for the different culture conditions. The PDAC cultures with TGF-β1, with an IC_50_ of 2.8 (±0.21), were more resistant to the effects of gemcitabine in comparison to PANC-1 cultures with TGF-β1 and the PDAC cultures without TGF-β1, with an IC_50_ of 1.9 (±0.07) and 2.5 (±0.14), respectively. Fig. 6E compares the percentage viability measured for the different culture conditions in Fig. 6D to their mechanical stiffness (Fig. 4). With a mechanical stiffness of 970 Pa for the PANC-1 cells in the presence of PSC and TGF-β1 (PDAC with TGF-β1), culture viability is decreased to about 60% with high gemcitabine concentration (250 µM).

## Discussion

PDAC is characterized by an extensive desmoplastic stroma, which is fundamental to the tumor tissue stiffness, disease aggression, and therapeutic resistance as it limits the delivery of chemotherapeutic drugs to the cancer cells [3], [5], [6], [9], [19], [23]. The PSC cells are responsible for the PDAC desmoplastic stroma by producing copious amounts of ECM, exacerbated by cytokines and signaling molecules, such as TGF-β [8], [9], [12], [13], [18], [21].

Previous studies show the fundamental role PSCs play in the development and growth of the fibrotic stroma and the effect of TGF-β on PDAC cellular behavior [12], [38], [40], [50], [51], but with few quantitative assessments on the mechanical stiffness of *in vitro* PDAC cultures, which include PSCs and TGF-β [23], [29], [31]. In assessing the ability to recapitulate the mechanical stiffness of the PDAC tumor tissue, a PDAC model consisting of PANC-1 and PSCs, with TGF-β growth supplement, was cultured for a period of 45 days, and their mechanical stiffness was assessed with oscillatory shear rheology.

### Effect of PSCs on PANC-1 spheroid growth

Prior to assessing the mechanical stiffness of the PDAC model, the presence of PSCs on the rate at which PANC-1 cultures grow was assessed by monitoring the diameter and doubling time of PANC-1 only spheroids and PDAC spheroids (Fig. 1, Fig. S1, Table S1). PSC only spheroids were observed to be dormant during culture as they did not show any significant growth (Fig. 1A), and this suggests the PSCs were quiescent without the presence of PANC-1 cancer cells [12], [13], [18], [21]. The PANC-1 only spheroid cultures were observed to increase in size, but their growth was not as significant as the PDAC spheroid cultures (Fig. 1B and C). This shows that PSC cells support and are important for PDAC cancer cell growth and tumor progression [12], [52], [53], [54].

Investigating the growth and size of the spheroids, regions of cells in different proliferative and metabolic states, as observed in solid tumors, were captured. Tumor spheroids have the unique capability of mimicking the physiological environment of tumors [34], [35], [45], [46], [47], and the spheroids were observed to have cells at the tumor periphery which were proliferative, followed by a layer of quiescent cells, and cells at the core which were dead (Figs. 2 and S2). The ability of tumor spheroids to mimic the physiological environment of solid tumors is dependent on their size. Typically, spheroids are able to model how tumors outgrow the supply of nutrients and oxygen available to them and the inability to remove metabolic by products as they grow and progress to more than 400–500 µm in diameter. Cells more than 100–200 µm from the tumor periphery receive less nutrients [35], [45], [46], [47]. Fig. S2 shows PDAC spheroids with a radius of 390 µm, 690 µm, and 1000 µm (day 7, 14, and 21 of culture, respectively), which gives a diffusion path for nutrients and oxygen greater than 150 µm, causing the different metabolic regions that can be seen by staining. *In vivo*, with the PSCs, ECM macromolecules accumulate, and together with the proliferation and accumulation of cancer and stromal cells, blood and lymphatic vessels collapse, resulting in a limited diffusion of nutrients, oxygen, and drugs to the cancer cells [12], [18], [21], [35], [45], [49].

### Tumor biophysical properties

The mechanical stiffness of the *in vitro* PDAC cultures, which include PSCs and TGF-β supplement, was assessed using oscillatory shear rheology (Fig. 3) for measurements of their storage (or elastic, *G′*) and loss (or viscous *G″*) moduli components (Figs. S4 and S5). As shear deformation was used to assess the mechanical properties of the cultures, the complex sum of their *G′* and *G″* measurements (the complex shear modulus, |G*|) determined their mechanical stiffness (Fig. 4), the extent to which they resisted shear deformation [43], [55].

PANC-1 only cultures had a mechanical stiffness of 23 Pa on day 45 of culture, but with the presence of the PSCs, the PDAC cultures had a mechanical stiffness of 650 Pa on day 45 of culture (Fig. 4). PSCs control the structural and functional integrity of the ECM in healthy and diseased pancreatic tissues. In PDAC, PSCs mediate the matrix environment by stimulating the synthesis, activity, and degradation of ECM macromolecules, with enzymes and proteases such as lysyloxidases (LOX), metalloproteinase (MMP) 2 and 9 and their tissue inhibitor of metalloproteinase (TIMPs) to generate a fibrotic stroma with large amounts of ECM that drives PDAC progression [3], [9], [10], [13], [22]. The large amounts of ECM macromolecules, such as fibrillar collagens and glycosaminoglycans with different mechanical properties, are reported to transmit or store stress, thus playing an integral role in the mechanical stiffness of the tumor tissue [3], [24], [25], [42]. Fibrillar collagens, which provides the basic framework of the ECM architecture, are stiff in tension and provides tensile strength to tissues as they absorb stress. Whereas, HA are gelatinous, which provide resistance to the stress with their ability to trap water and swell [17], [19], [25], [42], [56], [57], [58]. And together with the accumulation of highly proliferative cells, the tumor tissue becomes dense and compact, increasing in stiffness. This was seen with the increase *G′* and *G″* moduli from the increase collagen deposition (Fig. 5), resulting in an increase mechanical stiffness, |G*| (Fig. 4) [3], [9], [28], [42], [59]. The decrease and increase in |G*| between day 5 and 14 (Fig. 4) was believed to be effects of the remodeling of the ECM by the cells, especially with the presence of PSCs and TGF-β [3], [9], [10], [13], [50].

### Effect of TGF-β1

TGF-β1 is a polypeptide of the TGF-β cytokine family, and its signaling pathway is fundamental in regulating many cellular functions such as cell growth and proliferation, differentiation, and apoptosis. However, in cancer cells, TGF-β signaling abnormally enhances these cellular processes, including the production of a dense, rigid ECM [3], [25], [50], [60], [61], [62], [63], [64]. This was evident from the mechanical stiffness and the presence of collagen for the PANC-1 only cultures and the PDAC cultures with TGF-β1 (Figs. 4, 5 and S11).

From day 30 of culture, the PANC-1 only cultures with TGF-β1 had a mechanical stiffness of >340 Pa (Fig. 4B) and displayed more collagen in the culture periphery (Fig. S11). However, the PDAC cultures with TGF-β1, reaching a mechanical stiffness close to the PDAC tumor tissue by day 21 of culture (970 Pa), and a mechanical stiffness of >1170 Pa from day 30 (Fig. 4C), exhibited collagen in the culture environment (Fig. 5). This supports that the presence of activated PSCs in the tumor environment leads to the overproduction of ECM macromolecules and is further enhanced by the presence of TGF-β toward a desmoplastic stroma [3], [6], [50], [60], [62].

In quantifying the mechanical behavior of the cultures over a timescale, measurements of their *G′* and *G″* property also gave insight into the matrix and cellular dynamics of the cultures [43], [44], [55]. The PANC-1 with PSCs and TGF-β exhibited an increase in elasticity and viscosity (Fig. S5), which has been reported to be due to the concentration, organization, and mechanical properties of the ECM macromolecules [3], [42], [65], [66], [67], [68]. Fibrillar collagen and glycoprotein elastin have been reported to be associated with ECM elasticity and stiffness, providing cells and tissues with the structure, strength, and extensibility they require to grow and develop [42], [65], [67]. Whereas, the viscosity of the ECM is reported to be likely due to the gel-fluid phase of the ECM with the gelatinous property of glycosaminoglycans, such as HA, and their propensity to absorb, trap water, and swell [42], [58], [67]. Together, the viscoelastic behavior of the PANC-1 with PSCs and TGF-β show the ability of the cells to sense, respond, and reciprocate to the structure and dynamics of the growing heterogeneous ECM and the mechanical forces they exert [3], [42], [65], [68], [69]. The increase rigidity of the cultures imply the ability of the cells to respond to the growing mechanical forces of their environment, and cell sense and respond to increasing rigidity of their environment via integrins [23], [26], [40], [65], [69], [70].

### Effect of mechanical stiffness on the efficacy of gemcitabine

Gemcitabine is a nucleoside analogue used as the standard of care for patients with locally advanced and metastatic PDAC [5], [71], [72]. As a nucleoside analogue, gemcitabine competes against deoxycytidine triphosphate in the deoxynucleotide triphosphate pool to be incorporated into DNA. This leads to faulty DNA synthesis and cancer cell death [71], [72], [73]. However, with a stiff matrix, which impedes the delivery of drugs to the cancer cells, PDAC is resistant to gemcitabine [54], [71], [72], [73], [74]. This was seen for the PANC-1 cultures with PSC and TGF-β.

The PANC-1 cultures with PSC and TGF-β, cultured for 21 days to achieve *in vivo* mechanical stiffness, was unaffected by gemcitabine in comparison to the PSC only and PANC-1 only cultures with and without TGF-β, and the PDAC cultures without TGF-β (Fig. 6B, D and E). In the presence of PSC and TGF-β, PANC-1 accumulated ECM macromolecules (collagen in its matrix environment, Fig. 5), making their culture environment dense and stiff (Fig. 4) to obstruct the delivery of gemcitabine to the cancer cells. With increasing ECM components, for example collagen, there is increasing crosslinking of fibers, or a meshwork of a fibrotic stroma, which accounts for the increasing stiffness, the collapse of vasculatures, the increase interstitial pressure, and the decrease the porous structures or the interstitial space of the microenvironment limiting interstitial flow with drugs [24], [28], [75], [76], [77], [78]. This rigid, dense, fibrotic stroma thus forms a physical barrier for the transport of drugs to the cancer cells, and therefore this accounts for the therapeutic resistance observed *in vivo* and shown in our PDAC cultures. Nevertheless, if gemcitabine could permeate through the culture and affect the cells, its activity would be inhibited by the hypoxic and acidic environment commonly observed with PDAC [49], [54], [79], [80], [81], [82]. The PDAC cultures were stained for hypoxia-inducible factor-1 alpha (HIF-1 alpha) to determine if they exhibited a hypoxic environment, as shown in Fig. 6C. HIF-1 alpha is an important transcriptional factor that plays a pivotal role in various cell functions, including cellular adaptation to hypoxia [49], [79], [81], [83], [84], [85]. In PDAC, with the cancer cells deprived of nutrients and oxygen (as seen with our cultures, Figs. 2, S2, and 6C), the cancer cells adapt, switching on genes such as HIF-1 alpha to mediate and promote pathways and metabolic responses, such as the anaerobic glycolysis, for survival. However, as a result of the inadequate supply of glucose, there is a high concentration of the glycolytic byproduct lactate in the microenvironment, which inactivates any chemotherapeutic effect [3], [25], [49], [81], [83], [84], [85], [86]. Taken together, these finding highlight PSC cells and TGF-β promote a dense and stiff tumor microenvironment, which promotes resistance to the effect of therapeutics.

## Conclusion

The presence of PSCs in *in vitro* PDAC cultures is critical to accurately modeling the PDAC tumor growth and mechanical stiffness. Previous studies show the importance of PSCs for the PDAC stroma, but most mechanical studies of the *in vitro* PDAC cultures are of PDAC cancer cells only and do not include PSCs or TGF-β supplements. Here, we have demonstrated that the PDAC tissue mechanical stiffness can be recapitulated with *in vitro* PDAC cultures, which includes PSCs and TGF-β. We highlight that matrix environment is crucial in producing a model system that adequately recapitulates the growth and mechanical behavior of the PDAC tissue for effective therapeutic assessment. The tumor stiffness is related to the resistance of the tumor to drug penetration, yet it is not widely accounted for in current models as therapeutic assessment of potential drugs is performed at the earlier stages of culture before mechanical maturity is reached. In order to develop drugs that are effective against PDAC and improve patient survival, it is vital to encompass the correct biophysical microenvironment in *in vitro* models.

## Experimental section

### Cell lines and culture

The human pancreatic ductal adenocarcinoma cell line, PANC-1 (ECACC 87092802) [87], was maintained in DMEM (Thermo Fisher Scientific), 10% FBS (Sigma Aldrich) supplemented with 1% Penicillin Streptomycin (P/S; Sigma-Aldrich) and 1% GlutaMAX (Thermo Fisher Scientific).

The human pancreatic stellate cells, PSCs, were maintained in stellate cell medium supplemented with 10% FBS, 1% stellate cell growth supplement and 1% P/S in culture flasks coated with Poly-L-Lysine. The PSCs and respective reagents were sourced from ScienCell Research Laboratories supplied by Caltag Medsystems Ltd.

The PANC-1 and PSCs were cultured under humidified conditions (95–99%) at 37 °C with 5% CO_2_, and were passaged with 1 mL TrypLE Express Enzyme (1×) no phenol red (Thermo Fisher Scientific) and used once ≥70% confluence was achieved. Passages between 4 and 6 for the PSCs were used in the cultures.

### PDAC spheroid culture with ultra-low attachment plates

Once the PANC-1 and PSCs were ≥70% confluent, the cells were harvested and seeded into Corning® 96-well clear round bottom ultra-low attachment (ULA) plates (Scientific Laboratory Supplies) for PSC only, PANC-1only, and PDAC (co-culture of the PANC-1 and PSC cells at a seeding ratio of 1:2 or 1:3 for biological relevance [88]) spheroid cultures at a seeding density of 250, 500 and 1000 cells per well. The spheroids were cultured with DMEM/10% FBS culture medium (refreshed once a week by monitoring the medium pH) under humidified conditions at 37 °C with 5% CO_2_, and daily bright field images were taken and analyzed using a MATLAB SpheroidSizer program [89] for size and volume assessment. The spheroid volume (mm^3^) estimates were then used in Eq. (1) below to determine the doubling time of the spheroids, which is defined as the time it takes for the volume of the spheroid to double in size.

(1) $$\mathrm{Doubling}\;time=\frac{\mathrm{time}*\ln\left(2\right)}{\ln\left(finalvolume/initialvolume\right)}$$

where *time* is in days, *final volume* (mm^3^) was the spheroid volume at the end of culture, and *initial volume* (mm^3^) was the volume 24 h after seeding when the cells had aggregated into a spheroid.

### PDAC spheroid culture with an ECM environment

1 × 10^6^ cells mL^−1^ of the PANC-1 and PSCs were seeded into 21.5 cm^2^ Nunclon Delta surface treated cell culture petri dishes (Thermo Fisher Scientific) with 6–9 mg mL^−1^ of Cultrex® Basement Membrane Extract (BME) gel (R&D systems, Bio-Techne), which includes laminin and type IV collagen, for PSC only, PANC-1 only, and PDAC (PANC-1 and PSCs co-culture in a 1:3 seeding ratio) spheroid cultures with an ECM environment, which will be referred to as PSC only, PANC-1 only, and PDAC cultures, respectively. Basement membrane extract was used for the 3D culturing of the cells as it is a commonly used hydrogel or scaffold for 3D cell culture, including pancreatic cancer cells [36], [37], [90], [91]. Basement membrane was chosen to allow the cells to naturally engineer an environment (as opposed to the use of a synthetic hydrogel, which often lacks the presence of structured proteins and fail to capture the biophysical structures and cues of the cellular microenvironment) [92], [93], [94]. The PSC only, PANC-1 only and PDAC cultures were grown with 10 ng mL^−1^ of TGF-β1 growth factor supplement (Sigma-Aldrich) [60], [95], [96] in DMEM/10% FBS culture medium under humidified conditions at 37 °C with 5% CO_2_ for mechanical stiffness assessment.

### Oscillatory shear rheology of PDAC cultures

Oscillatory shear rheology was used to measure the mechanical stiffness of the PANC-1 only and PDAC cultures with and without TGF-β1. Oscillatory shear rheology measures the response exhibited by samples in resisting a change in shape under oscillatory shear deformation. It assesses the sample’s elastic or storage (*G′*) and viscous or loss (*G″*) moduli components [32], [43], [44], [55]. With these measurements, the complex shear modulus (G*), is used to determine the sample's mechanical stiffness, the extent to which the sample is able to resist deformation [32], [43], [44], [55]. Eq. (2) below defines the complex shear modulus with the storage and loss moduli components.

(2) $$G^{*}=G^{\text{'}}+{\mathrm{iG}}^{\text{'}\text{'}}$$

where *G′* is the storage modulus, *G″* is the loss modulus, and G* (or |G*|) is the complex shear modulus, which is the quadrature summation or the complex sum of *G′* and *G″* moduli. All units are in Pascal (Pa).

The storage modulus component, *G′*, is the energy stored by the sample from a change in shape under shear deformation, recoverable when the deformation form is removed, and the loss modulus component, *G″*, is the energy lost or used when the sample is recovering into its original shape. However, biological samples are neither purely elastic nor purely viscous due to their molecular arrangements, and the complex sum of their *G′* and *G″* components, |G*|, provides information on their mechanical stiffness [3], [32], [42], [43], [44], [55].

Fig. 3 shows the set up for the mechanical stiffness assessment of the cultures with oscillatory shear deformation. Replicate cultures of the PANC-1 and PSC cells seeded into cultures dishes with BME gel, grown for 45 days with and without TGF-β1, from three separate seeding settings, were used for the assessment. For shear deformation assessment, the cultures were taken from the incubator and placed on the bottom plate of an Anton Paar MCR 302 stress-controlled rheometer. A 50 mm parallel plate, with anti-slip material to prevent sample slippage on the surface [32], was brought into contact with the cultures at working gap of 0.8 mm. Pseudo-strain controlled shear deformation time sweeps were performed at a shear strain of 2% and a frequency of 0.5 Hz for 600 s. As the gap was set to 0.8 mm, a range of normal forces was measured to ensure contact between the parallel plate and the culture samples. Frequency sweep measurements from 10 Hz to 0.01 Hz, with a constant shear strain of 2%, were also performed to determine the long-term structural stability of the cultures under deformation. After shear deformation, the parallel plate geometry was released from contact with the cultures, and the cultures were taken off the bottom plate for disposal. From the shear deformation time sweep measurements, *G′* and *G″* components of the culture samples were determined by averaging their steady state measurements over the measurement time. |G*| was then calculated using Eq. (2) to determine the mechanical stiffness of the cultures.

Eq. (2) was used to determine |G*| of the cultures, and the Youngs modulus (*E*) of the cultures was determined using.

(3) E=2G(1+v)

where *E* is the Youngs modulus in Pa, *G* is the complex shear modulus, |G*|, derived using Eq. (2), and *v* is the Poisson ratio (approximated as 0.5) [97].

Moreover, daily bright field images of the cultures were taken to assess the effects of the presence of PSC and TGF-β and to determine if the mechanical stiffness was due to an increase in cell number, size, or increase in the number of spheroids. Using the Binary function in ImageJ, the effect of cell number, size, and or spheroid number on the mechanical stiffness of the cultures, was determined.

### Immunostaining of collagen, actin and HIF-1 alpha

The different culture conditions, PSC only cultures, PANC-1 only cultures, and PDAC (PANC-1 and PSCs co-culture in a 1:3 seeding ratio) cultures with and without TGF-β1, were fixed with Formaldehyde 4% aqueous solution (VWR; kindly provided by Dr Zhang Y. Ong) for 30 min at room temperature, and then permeabilized with 0.2% Triton X-100 (Sigma Aldrich) in 1% BSA (Sigma Aldrich), 5% FBS (Sigma Aldrich) solution for 1 h at room temperature. The cultures were then stained with recombinant anti-collagen I antibody, recombinant anti-HIF 1 alpha antibody overnight at 4 °C. Secondary staining was performed with goat anti-rabbit Alexa Fluor™ 488 for 1 h at room temperature. The primary, collagen and HIF-1 alpha, and secondary antibodies were prepared in 1 mL PBS containing 1% BSA. Actin was stained with phalloidin-iFluor 647. The antibodies and actin stain were all sourced from Abcam. Nuclei were stained with DAPI (Boster Biological Technology). The cultures were then imaged with a Leica confocal fluorescence using a 10× objective and a pinhole of 1.00 AU with the respective excitation and emission wavelength of the stains according to the manufacturer’s instructions. The acquired images were analyzed with Image J.

### Gemcitabine assessment

Gemcitabine was purchased from Sigma and dissolved into a 5 mg mL^−1^ stock solution in DMSO (Sigma Aldrich) and stored at −20 °C until use. The stock was then diluted with DMEM/10% FBS solution to treat the cultures and assess its effect. The PSC only, PANC-1 only, and PDAC (co-culture of the PANC-1 and PSC cells in a seeding ratio of 1:3) spheroids at a seeding density of 250 cells per well in the ULA plates (no BME), cultured with and without TGF-β1 for 21 days to achieve mechanical stiffness, were treated with different concentrations of gemcitabine between 0.09 and 500 µM in a 2-fold dilution. The PSC only, PANC-1 only, and PDAC (co-culture of the PANC-1 and PSC cells in a seeding ratio of 1:3) cultures, with the cells seeded at 1 × 10^6^ cells mL^−1^ in the culture petri dishes with 6–9 mg mL^−1^ BME and cultured with and without TGF-β1 for 21 days, were treated with 7.8, 31.25 and 250 µM of gemcitabine. 72 h after exposing the spheroids and cultures with the different gemcitabine concentrations, viability was assessed by quantifying their ATP content with CellTiter-Glo® 3D cell viability assay (Promega). The viabilities were normalized to their respective positive and negative controls to determine the effect of gemcitabine.

### Viability assessment with live/dead assay

The spheroids were stained and incubated with 2 µM Calcein AM, 4 µM Ethidium homodimer-1 (EthD-1) and 5 µg mL^−1^ of Hoechst 33,342 at 37 °C with 5% CO_2_ according to the manufacturer’s instructions. All three stains were sourced from Thermo Fisher Scientific. After incubation, the spheroids were imaged with the Leica confocal fluorescence using a 10× objective and pinhole of 1.00 AU with the respective excitation and emission wavelengths of the stains according to the manufacturer’s instructions. The acquired images were analyzed with Image J.

### Viability assessment with ATP viability assay

The ATP viability of the cultures, with and without exposure to gemcitabine, was assessed by measuring their metabolic activity with the CellTiter-Glo reagent. ATP is a marker for the presence of metabolically active cells. A volume of CellTiter-Glo® 3D reagent, equal to the volume of culture media present in the wells of the ULA plate or culture dishes, was added for luminescence reading with a microplate reader (SpectraMAX M2, Molecular Devices)*.*

### Statistical analyses

Data were expressed as the mean ± standard error (SE), and using OriginPro software, statistical significance was assessed with paired or two sample *t*-test or two-way ANOVA followed by pairwise comparison with Tukey’s multiple comparison test, where a *p* ≤ 0.05 was considered statistically significant.

## Author contributions

D.K designed, performed the experiments, and analyzed the data with input from all authors. D.K. and M.D.G.H designed the oscillatory shear rheology experiments and acquired the data. D.K and S.A.P wrote the manuscript with input from all authors.

## Declaration of Competing Interest

The authors declare that they have no known competing financial interests or personal relationships that could have appeared to influence the work reported in this paper.
