# Supplementary material for: Modeling the mechanical stiffness of pancreatic ductal adenocarcinoma
Source: Matrix Biol Plus. 2022 Mar 21;14:100109. doi: 10.1016/j.mbplus.2022.100109 (PMC8990173; doi:10.1016/j.mbplus.2022.100109)
Supplement: Supplementary data 1 [file mmc1.docx]

Supplementary Data

Modeling the Mechanical Stiffness of Pancreatic Ductal Adenocarcinoma

Delanyo Kpeglo, Matthew D. G. Hughes, Lorna Dougan, Malcolm Haddrick, Margaret A. Knowles, Stephen D. Evans, and Sally A. Peyman*

* Correspondence to Dr. S. A. Peyman, [S.Peyman@leeds.ac.uk](mailto:S.Peyman@leeds.ac.uk)

B)

A)

**Figure S1.** The size in width of PDAC spheroids cultured for 21 days. A) of PDAC spheroids cultured without TGF-β1, and B) of PSC only, PANC-1 only, and PDAC spheroid cultures with and without TGF-β1 (10 ng mL^-1^) growth factor supplement. Seeding density of 250 cells per well. For the PDAC cultures, seeding ratio between PANC-1 and PSC cells is 1: 3.

**Table S1.** The doubling time of PDAC spheroids cultured for 14 or 21 days. *n* = 3 or 9 spheroids

| Seeding density | Seeding ratio of PANC-1 and PSC cells | Cultured for [in days] | Mean volume [mm^3^] on day 1 | Mean volume [mm^3^] on day 14 or 21 | Mean doubling time [in days] |
| --- | --- | --- | --- | --- | --- |
| 250 | 1 : 3 | 21 | 0.005 ± 5.4E-04 | 0.62 ± 0.039 | 2.91 ± 0.06 |
| 500 | 1 : 3 | 21 | 0.009 ± 0.002 | 0.84 ± 0.097 | 3.08 ± 0.07 |
| 500 | 1 : 2 | 14 | 0.017 ± 0.005 | 0.37 ± 0.010 | 2.91 ± 0.29 |
| 1000 | 1 : 2 | 14 | 0.034 ± 0.003 | 0.50 ± 0.019 | 3.34 ± 0.13 |




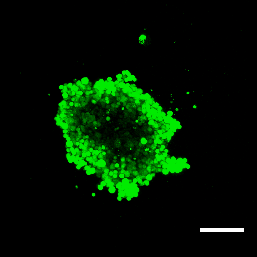

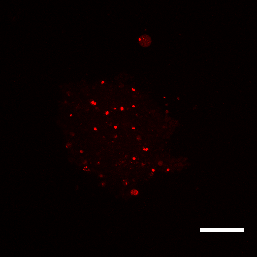

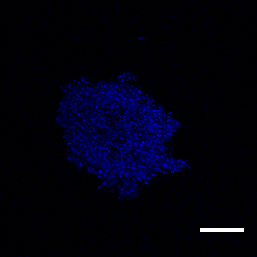

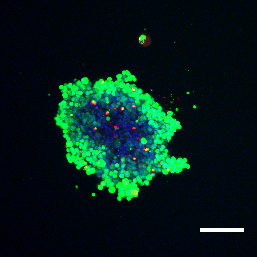

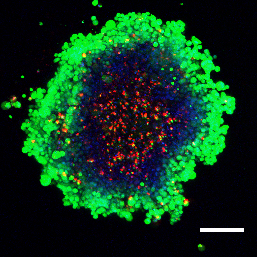

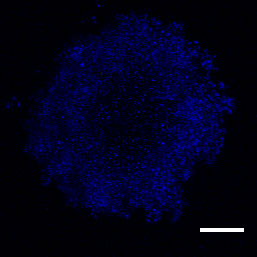

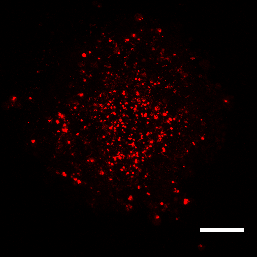

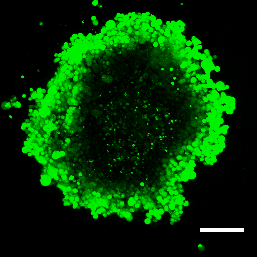

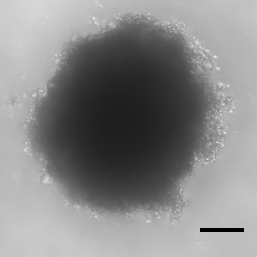

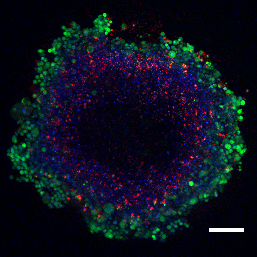

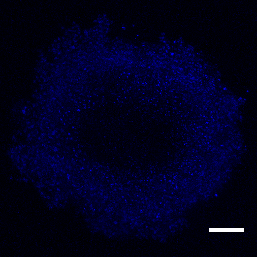

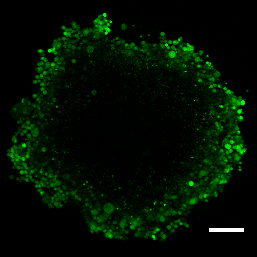

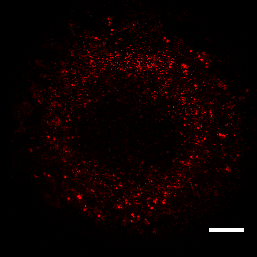

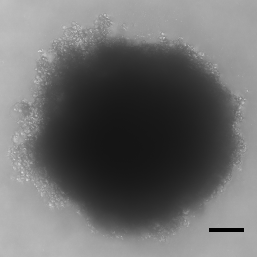


Hoechst

Calcein AM

EthD-1

Bright field

Merged

Day 7

Day 14

Day 21

**Figure S2.** The live/dead viability assessment of the PDAC spheroids (1:3 seeding ratio between PANC-1 and PSC cells) cultured for 21 days. Scale bar, 200 µm.

**Figure S3.** Metabolic activity of the PDAC (1:3 seeding ratio between PANC-1 and PSC cells) spheroids.

PDAC with TGF-β1 supplement

PDAC without TGF-β1 supplement

**Figure S4.** Examples of the *G*’ and *G*’’ moduli measurements of PDAC cultures with and without TGF-β1 on day 0 (3-4 hours after seeding cells), 5, 7, and day 45 of culture, of which an average of their steady state measurement over time (red dash arrow line in the box magnification on day 0 and day 45 as examples), is used to determine their |*G**|.

B)

A)

**Figure S5.** A) Storage or elastic (*G*’) and B) loss or viscous (*G*’’) moduli components of PDAC cultures with and without TGF-β1 growth factor supplement.

**Figure S6.** Range of normal forces measured by the parallel plate rotary system to ensure contact with the cultures during shear deformation.

**Figure S7.** Ratio of cells to gel in the PSC only, PANC-1 only, and PDAC cultures with and without TGF-β1 for mechanical stiffness assessment.

PDAC without TGF-β1 supplement

PDAC with TGF-β1 supplement

**Figure S8.** Examples of the frequency sweep measurements of the *G*’ and *G*’’ moduli components of PDAC cultures with and without TGF-β1 on day 0 (3-4 hours after seeding cells), 5, 7, and day 45 of culture at 2% shear strain.

A)

C)

B)

**Figure S9.** A) |*G**|, B) *G*’ and *G*’’ moduli, and C) frequency measurements of 6 – 9 mg mL^-1^ of BME gel over a 21-day period*.*


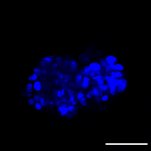

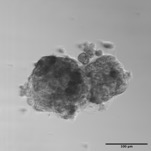

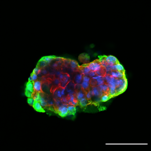

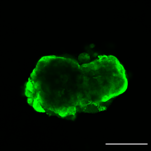

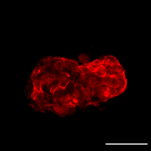

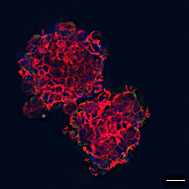

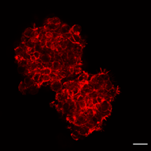

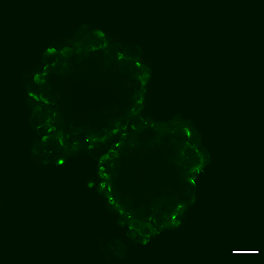

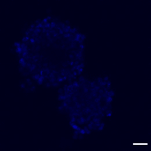

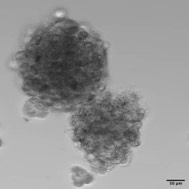

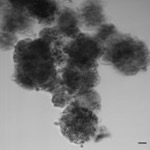

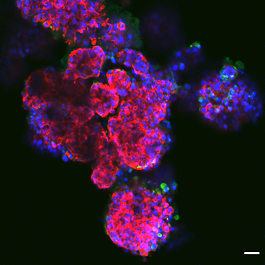

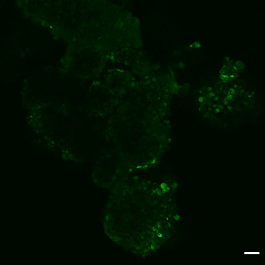

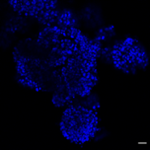

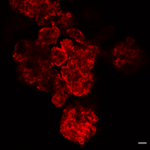

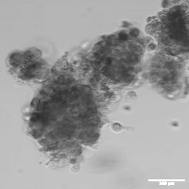

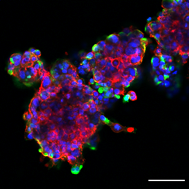

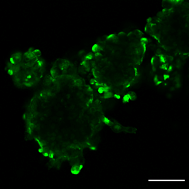

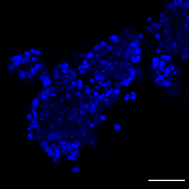

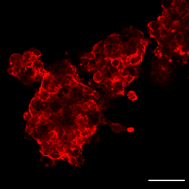


DAPI

Collagen

Phalloidin

Bright field

Merged

Day 7

Day 14

Day 21

Day 30

**Figure S10.** Immunofluorescence stain of collagen type I of the PANC-1 only cultures without TGF-β1 on day 7, 14, 21 and 30 of culture. Nuclei stained with DAPI in blue, collagen in green, actin with phalloidin in red. Scale bar on day 7 and 14, 50 µm. Scale bar on day 21 and 30 of culture, 100 µm,


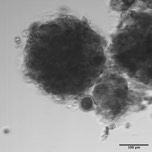

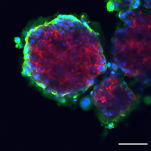

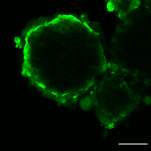

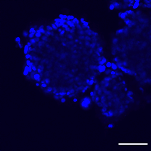

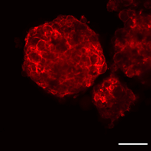

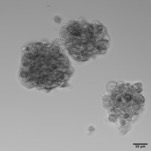

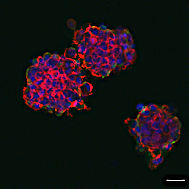

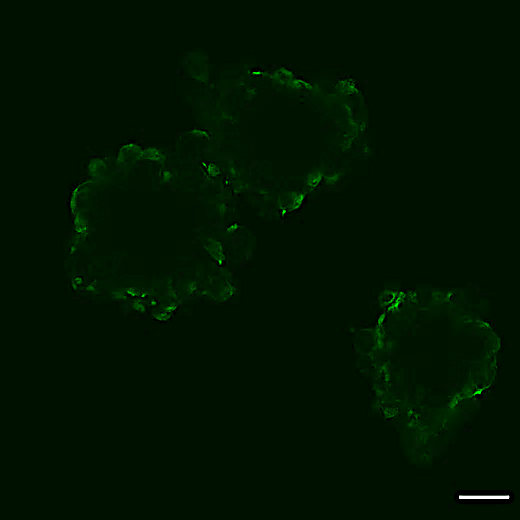

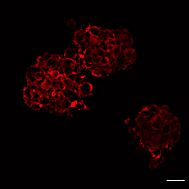

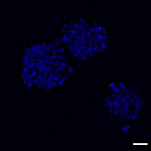

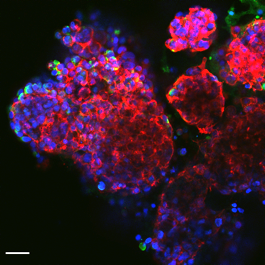

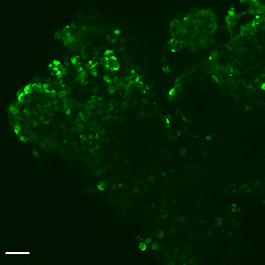

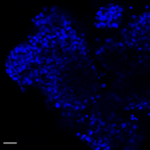

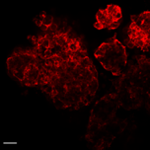

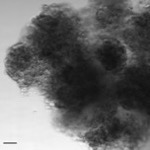

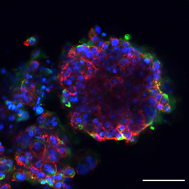

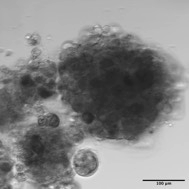

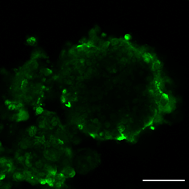

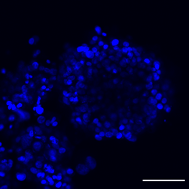

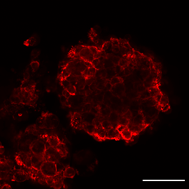


DAPI

Collagen

Phalloidin

Bright field

Merged

Day 7

Day 14

Day 21

Day 30

**Figure S11.** Immunofluorescence stain of collagen type I of the PANC-1 only cultures with TGF-β1 on day 7, 14, 21 and 30 of culture. Nuclei stained with DAPI in blue, collagen in green, actin with phalloidin in red. Scale bar on day 7 and 14, 50 µm. Scale bar on day 21 and 30 of culture, 100 µm,


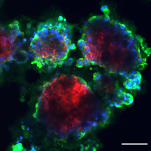

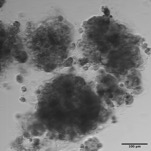

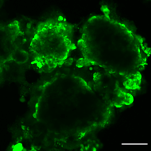

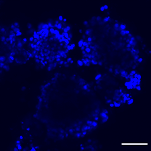

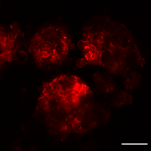

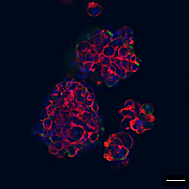

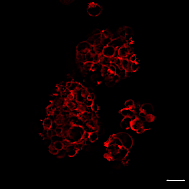

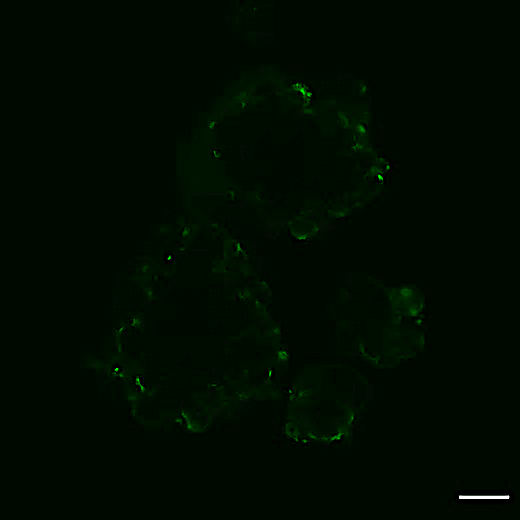

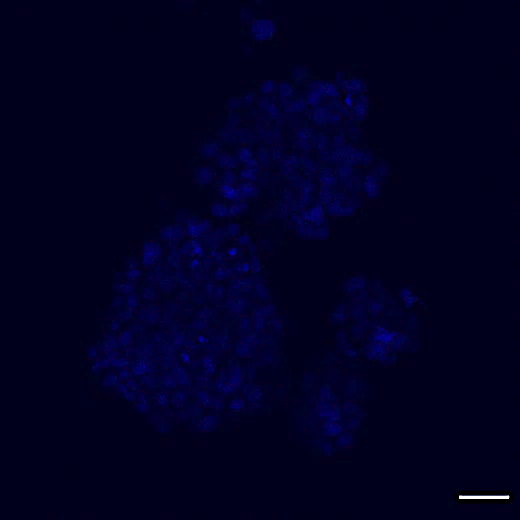

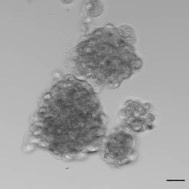

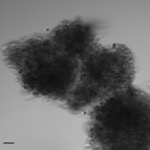

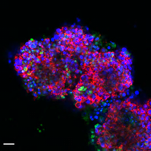

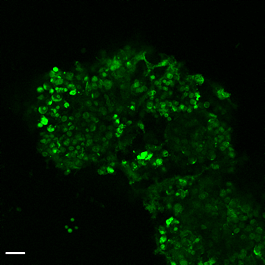

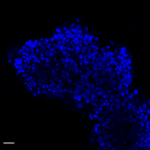

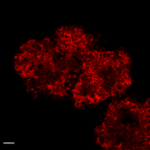

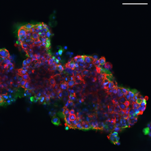

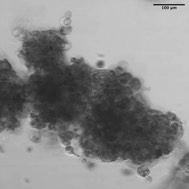

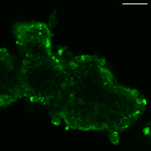

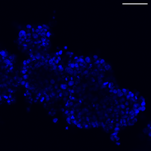

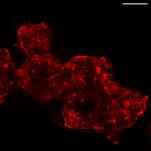


DAPI

Collagen

Phalloidin

Bright field

Merged

Day 7

Day 14

Day 21

Day 30

**Figure S12.** Immunofluorescence stain of collagen type I of the PDAC (PANC-1 and PSC co-culture at a seeding ratio of 1: 3) cultures without TGF-β1 on day 7, 14, 21 and 30 of culture. Nuclei stained with DAPI in blue, collagen in green, actin with phalloidin in red. Scale bar on day 7 and 14, 50 µm. Scale bar on day 21 and 30 of culture, 100 µm.
